# Sensitivity and Scenario Analysis to Reduce the Carbon Footprint of Polypropylene Processing Using Primary Industrial Data

**DOI:** 10.3390/polym18141760

**Published:** 2026-07-18

**Authors:** Chiara Antonacci, Elena Battiston, Diego Zamboni, Silvia Gross, Anna Mazzi

**Affiliations:** 1 Department of Industrial Engineering, University of Padua, 35132 Padua, Italy; 2 Department of Chemical Sciences, University of Padua, 35131 Padua, Italysilvia.gross@unipd.it (S.G.)

**Keywords:** carbon footprint, polypropylene, life cycle assessment, plastic processing

## Abstract

Life cycle assessment (LCA) studies of polypropylene (PP) processing commonly rely on generic secondary databases, while primary industrial inventories for plastic conversion processes remain scarce. This study addresses this gap by quantifying the cradle-to-gate carbon footprint of polypropylene processing using anonymised primary industrial data collected in 2024 from four European polypropylene processing facilities. Unlike previous studies relying mainly on generic secondary inventories, the proposed approach combines primary industrial data with sensitivity and scenario analyses to identify practical priorities for emission reduction. The baseline carbon footprint was estimated at 1.44 tCO_2_e per tonne of finished product, with material production and energy-intensive processing identified as the major emission hotspots. One-Factor-at-a-Time (OFAT) sensitivity analysis showed that polypropylene type, process efficiency, renewable electricity use, and process waste management were the most influential parameters, whereas water consumption and additive use had only a minor effect on overall emissions. Scenario analysis indicated that combining recycled polypropylene, improved process efficiency and renewable electricity reduced emissions by 45.8%, while reducing process waste and fully recycling production residues achieved a 42.2% reduction compared with the baseline. By integrating primary industrial inventory data with sensitivity and scenario analyses, this study provides a more representative assessment of real industrial polypropylene processing conditions than approaches based solely on generic databases and identifies practical priorities for industrial carbon mitigation.

## 1. Introduction

In recent years, global plastic production has steadily increased, exceeding 430 million tons in 2024 [1]. This growth reflects the central role that these materials play across many industrial sectors. Plastics are widely used in products, manufacturing processes, and packaging due to their relatively low cost and their properties of lightness, versatility, and durability [2]. However, their increasing use is also associated with significant environmental impacts. In particular, plastic production contributes substantially to the overall environmental burden, mainly due to the high energy intensity of production processes and the continued reliance on fossil-based raw materials [3]. These issues have led to growing interest in the scientific literature in assessing the environmental impacts associated with plastic materials and their production processes. In this context, Life Cycle Assessment (LCA), a standardized methodology for evaluating environmental impacts across the life cycle of products and processes, is one of the most widely used tools to quantify them [4].

Recent studies have highlighted significant methodological challenges and data gaps in LCA studies on plastics, particularly regarding the availability and representativeness of inventory data for industrial processing stages [5]. This limitation is especially relevant because the processing phase represents the segment of the value chain over which manufacturers generally have the greatest degree of operational control [6]. Parameters such as machinery energy efficiency, process yields, scrap rates, and input material composition can significantly influence the carbon footprint of final products, yet they are rarely analysed in a systematic and quantitative way [7]. As a result, many studies rely on average data or simplified inventories that do not adequately capture the variability of real industrial processes. This reduces the representativeness of the results and limits their usefulness for companies aiming to develop effective emission reduction strategies [5].

To address this gap, the collection of primary process-level data according to established standards allows for more reliable estimates of emissions associated with industrial processes. In this context, ISO 14064-1 [8] provides a recognized framework for the quantification and reporting of greenhouse gas emissions. Process-based analyses, which focus on emissions from individual production processes, make it possible to identify key emission hotspots and to generate reliable datasets that can support both industrial decarbonisation strategies and improvements in the quality of inventories used in Life Cycle Assessment studies. Among thermoplastic polymers, polypropylene (PP) is one of the most widely used materials worldwide due to its low cost, good chemical and mechanical resistance, and high versatility. It is extensively applied in several industrial sectors, including packaging, automotive, medical devices, and consumer products [9].

Unlike previous cradle-to-gate LCA studies of polypropylene processing, which predominantly rely on generic secondary databases or industry-average inventories, the present study is based on primary industrial data collected from four operating European polypropylene processing facilities. By integrating site-level industrial inventories with sensitivity and scenario analyses, the proposed approach not only provides a more realistic assessment of current industrial practice but also identifies the relative importance of the main operational parameters driving greenhouse gas emissions. This study contributes to bridging the gap between generic LCA inventories and real industrial decision-making by providing evidence-based priorities for emission reduction under realistic operating conditions. The study was designed to address the following research questions:

(i) What are the main contributors to the cradle-to-gate carbon footprint of industrial polypropylene processing?
(ii) Which operational parameters have the greatest influence on greenhouse gas emissions?
(iii) Which technically feasible mitigation strategies offer the greatest potential for reducing the carbon footprint of polypropylene processing?

To answer these questions, a cradle-to-gate Life Cycle Assessment was performed using primary industrial inventory data collected from four European polypropylene processing facilities, followed by One-Factor-at-a-Time sensitivity analysis and integrated mitigation scenario evaluation.

## 2. Materials and Methods

### 2.1. Goal and Scope Definition

The objective of this study is to evaluate potential strategies for reducing the carbon footprint associated with polypropylene processing, used as a representative case study for plastic materials. The analysis focuses on the industrial processing stage of the material, with the aim of identifying the main operational parameters that influence greenhouse gas emissions generated during the production process. The functional unit is defined as 1 ton of finished polypropylene plastic product. The study adopts a cradle-to-gate approach, including all stages from raw material production to the manufacturing of the final plastic product. In particular, the system boundaries include the stages of material treatment, extrusion, mixing, and injection moulding. The product use phase and the end-of-life stage of the final product are not included within the system boundaries. The exclusion of these phases is justified by the objective of the study, which is to provide an in-depth analysis of the upstream and core stages of industrial processing, where companies typically have the highest degree of operational control and which are less documented in the scientific literature compared to the use phase and plastic waste management, already widely analysed in Life Cycle Assessment studies [7,10]. In this study, the term “end-of-life” refers exclusively to the management of process waste generated during the transformation stages, which is included within the system boundaries, and not to the disposal or recycling of the final plastic product after its use.

### 2.2. System Description and Process Flow

**Figure 1:**
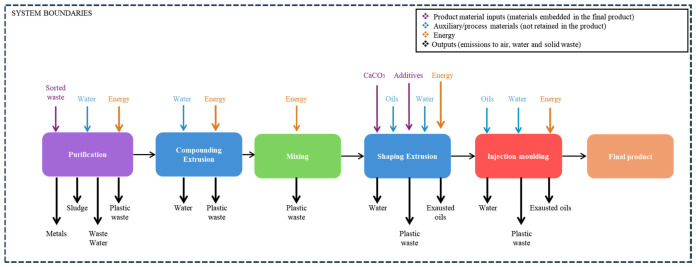
Supply chain flow diagram of polypropylene processing.

Based on primary data collected directly from four companies operating in the polypropylene processing sector during the 2024 production year, a representative process model was developed to reflect typical industrial conditions. The dataset comprises four industrial production lines, including three companies located in Italy and one in Austria, representing both large industrial facilities and small- and medium-sized enterprises. To ensure comparability, all input and output flows were normalised to the functional unit prior to aggregation. The resulting inventory was obtained by averaging the normalised data from the four production lines and therefore represents typical polypropylene processing operations rather than any individual facility. This aggregation approach improves the robustness of the dataset while preserving the confidentiality of the participating companies. The system flow diagram considered in the study is represented in Figure 1, and the main process stages considered in the study are the following:

- Material purification: mixed plastic waste is treated to remove metals, sludge, and other impurities. Outputs include purified material sent to extrusion and residual waste fractions.
- Compound extrusion: the purified material is extruded to produce polypropylene pellets.
- Mixing: the pellets are mixed to ensure homogeneity and to allow complete cooling before the subsequent processing stages.
- Shaping extrusion: the pellets are extruded to produce a semi-finished plastic material intended for injection moulding.
- Injection moulding: the semi-finished material is transformed into the final product through an injection moulding process.

The dataset does not include other polypropylene processing technologies, such as blow moulding, thermoforming, rotational moulding, compression moulding, fibre spinning, film stretching, foaming processes or additive manufacturing. Similarly, downstream product assembly, packaging, distribution, and product use were excluded from the system boundaries. Consequently, the conclusions should be interpreted as applicable only to polypropylene processing routes characterised by extrusion-based operations followed by injection moulding.

### 2.3. Data Collection and Data Treatment

The primary data used in this study were collected under confidentiality agreements with the companies participating in the study within a funded project. For this reason, detailed information at the individual company level cannot be disclosed. The available datasets refer to different configurations and technologies for plastic processing and were provided in an anonymized and aggregated form. To ensure system consistency, all input and output flows were normalized to the functional unit of 1 ton of finished product. When multiple datasets were available for the same process stage, inventory data were aggregated by calculating mean values, aiming to represent an intermediate industrial configuration. This approach provides a realistic description of typical industrial processing conditions while preserving data confidentiality. Although using average values limits the analysis of site-specific performance, it enables the identification of general trends and priority areas for improvement, in line with the objectives of sensitivity and scenario analyses.

The industrial dataset used in this study was derived from four polypropylene processing facilities, including three plants located in Italy and one in Austria. Although these companies represent different production capacities, ranging from small and medium-sized enterprises to large industrial facilities, the sample cannot be considered statistically representative of the entire European or global polypropylene processing sector.

The geographical scope of the dataset may influence the transferability of the results, particularly with respect to electricity supply and industrial operating conditions. Electricity-related emissions are representative of the Italian electricity mix adopted in the assessment and may therefore differ from those expected in regions characterised by lower- or higher-carbon electricity systems. In addition, electricity mixes evolve over time according to changes in national energy policies and the increasing adoption of renewable energy sources.

Waste treatment was modelled using secondary emission factors from authoritative sources rather than site-specific inventories. Whenever available, Italian datasets (e.g., wastewater treatment, sludge management, and waste oils from ISPRA) were adopted, while internationally recognised EPA emission factors were used for plastic residues and metals due to the lack of comparable national datasets.

Therefore, the reported carbon footprint values should be interpreted as specific to the investigated industrial configurations rather than as fixed reference values for polypropylene processing. The primary objective of this work is not to derive universally applicable carbon footprint values for polypropylene processing, but to identify the dominant contributors to the cradle-to-gate carbon footprint, determine which operational parameters most strongly influence greenhouse gas emissions, and evaluate realistic strategies for reducing emissions using primary industrial data.

### 2.4. Life Cycle Modelling

The study was conducted in accordance with the principles and requirements of ISO 14040 [11] and ISO 14044 [12]. A life cycle inventory was developed based on primary industrial data and complemented with secondary data from authoritative and open-access sources. The assessment focuses on climate change impacts, expressed as the carbon footprint in terms of CO_2_-equivalent emissions. Emission sources were classified according to the categories defined by ISO 14064-1 [8] to ensure consistency with common organisational greenhouse gas accounting practices.

Specifically, the model includes the following categories:

- Indirect emissions from purchased electricity (ISO 14064-1 [8], Category 2);
- Indirect emissions from purchased goods and services, including materials, additives, and auxiliary materials (ISO 14064-1 [8], Category 4.1);
- Indirect emissions from waste treatment, including the management of process waste generated within the system boundaries (ISO 14064-1 [8], Category 4.3).

Other emission categories defined by ISO 14064-1 [8] were not included in the analysis due to the lack of available company-level data. Consequently, this assessment does not represent a complete corporate carbon footprint but rather a targeted evaluation of production processes, aiming to quantify the most relevant and measurable emission sources within the processing stages considered.

This approach provides operational insights for companies, including:

- Identification of the most emission-intensive process stages;
- Quantification of the relative contribution of energy, materials, and waste management to total emissions;
- Assessment of potential emission reduction strategies in polypropylene processing.

Moreover, the availability of primary process-level emission data can help improve the quality of inventories used in Life Cycle Assessment studies, providing data that are more representative of actual operating conditions compared to generic datasets available in LCA databases.

Greenhouse gas emissions expressed in CO_2_-equivalents were calculated as the sum of the products of activity data and the corresponding emission factors:

Total emissions = ∑[(Ai × EFi)](1)

where Ai represents the activity data for each flow (materials, energy, water, or waste) and EFi the corresponding emission factor. The emission factors reported in Table 1 are expressed in CO_2_-equivalent units based on the 100-year Global Warming Potential (GWP100) metric adopted by the original sources [13]. Emission factors were selected from authoritative and open-access sources, in line with ISO 14064-1 [8] recommendations. When available, preference was given to datasets representative of the Italian or European context to improve the geographical representativeness of the results.

Among the considered input flows, additives represent one of the most uncertain inventory components due to the limited availability and transparency of publicly accessible life cycle inventory data, which are often incomplete or not representative of actual industrial formulations [10]. Although the participating companies provided primary data on the amount of additives used in each production process, the chemical composition of the additives was not disclosed because the formulations are proprietary. Consequently, formulation-specific life cycle inventories and emission factors could not be assigned.

To ensure that additive-related emissions were not omitted from the assessment, a proxy emission factor was adopted based on the cradle-to-gate Environmental Product Declaration (EPD) of a commercial polypropylene masterbatch developed for extrusion and injection moulding applications [14]. This product was selected because its intended use is consistent with the processing routes investigated in this study and because no publicly available life cycle inventories were available for the proprietary additive formulations used by the participating companies.

Although the selected masterbatch does not represent the full range of additive chemistries used in polypropylene compounding, it was considered a pragmatic and conservative proxy that enables additive-related emissions to be explicitly represented in the inventory rather than omitted entirely. This approach is also in line with the limitations associated with plastic additive inventories reported in the literature [10]. Accordingly, the adopted proxy should be interpreted as a practical solution to address the lack of formulation-specific data rather than as a representation of the actual environmental profile of the proprietary additives used by the participating companies. All emission factor sources adopted in the life cycle inventory are reported in Table 1.

In the analysed production systems, additives accounted for approximately 11 kg per tonne of finished polypropylene (about 1.1 wt%). Therefore, although different additive formulations may exhibit different environmental profiles, their relatively small mass fraction limits their contribution to the overall carbon footprint compared with the polymer matrix and electricity consumption.

**Table 1:** Sources of emission factors used in the inventory.

Category	Description	Value [kgCO_2_e/kWh]	Source
2.1	Energy (Italian mix 2023)	0.2363	ISPRA [15]
Category	Description	Value [tCO_2_e/t]	Source
4.1	Polypropylene (virgin)	1.632	PlasticEurope (2022) [16]
	Sorted plastic waste	0.015	EIB [17]
	CaCO_3_	0.1	EC-JRC (2010) [18]
	Mineral oils	1.40	UK Gov.—DEFRA [19]
	Additives	1.25	Lignin Industries AB (2023) [14]
	Water supply	0.0001913	UK Gov.—DEFRA [19]
4.3	Water treatment	0.000171	ISPRA [15]
	Sludge	0.729	ISPRA NID 2025 [20]
	Plastic residual (recycle)	0.220	EPA [21]
	Plastic residual (combusted)	3.086	EPA [21]
	Plastic residual (landfill)	0.022	EPA [21]
	Metals (recycled)	0.253	EPA [21]
	Metals (landfilled)	0.022	EPA [21]
	Metals (combusted)	0.011	EPA [21]
	Exhausted oils	0.005	ISPRA [15]

### 2.5. Sensitivity Analysis

Sensitivity analysis allows the evaluation of how variations in input parameters affect the estimated environmental impacts, enabling the identification of the variables that exert the greatest influence on model results and the assessment of the robustness of the conclusions. This approach makes it possible to identify variables with the highest emission reduction potential and to support the definition of improvement strategies for polypropylene processing. A One-Factor-at-a-Time (OFAT) approach was adopted, in which a single parameter is varied at each iteration while all others are kept constant at their baseline values. A One-Factor-at-a-Time sensitivity analysis was selected because the primary objective of this study was to quantify the individual influence of each operational parameter on the overall carbon footprint while maintaining the remaining parameters constant. This approach facilitates the interpretation of individual parameter contributions and enables the identification of priority intervention areas for industrial decision-making. Although global sensitivity methods can account for parameter interactions, they require detailed probability distributions and correlation structures that were not available. Therefore, OFAT was considered the most appropriate and transparent approach for the available data. This approach is commonly used in sensitivity analysis studies to transparently assess the influence of individual parameters on model results [22]. The parameter groups were selected based on the main factors identified in the literature as influencing greenhouse gas emissions in plastic processing, including the type of raw material used, the energy consumption of extrusion and moulding processes, the use of additives, and the management of production waste [7,10]. The baseline configuration is based on recycled polypropylene, reflecting the availability of primary industrial data for this production route. The analysed system represents a real industrial process involving sorting, purification, extrusion, and injection moulding. In contrast, virgin polypropylene was modelled using secondary data from literature and databases. This choice ensures consistency and reliability of the baseline scenario while allowing comparison with alternative material configurations.

The sensitivity analysis considered variations in the following parameter groups:

- Origin of polypropylene (virgin, recycled, or blends of both), as the use of recycled polymers is widely recognized as a key factor influencing the environmental performance of plastic production systems [7];
- Operational efficiency and process performance; based on primary industrial data, best-case and worst-case configurations were identified to represent the range of variability in process performance. These configurations reflect differences in key operational parameters, including energy consumption, material losses, and process efficiency observed across the analysed production lines;
- Electricity supply mix, which strongly affects the environmental impacts of energy-intensive industrial processes [1];
- Use of additives, which may influence the environmental profile of polymer products depending on their composition and associated production processes [7];
- Process energy consumption, as extrusion and moulding are typically among the most energy-intensive stages in polymer production [3];
- Water consumption, which may affect environmental impacts depending on process operating conditions;
- Amount of process waste generated, as variations in material yield can significantly affect resource use and waste generation in manufacturing systems;
- End-of-life management options for process waste, as different treatment scenarios can significantly influence life cycle impact results.

The magnitude of variations applied to each parameter was defined to represent plausible changes, based on values reported in the literature and typical operating ranges of industrial plastic processing. For each sensitivity case, the life cycle inventory was updated, CO_2_-equivalent emissions were recalculated using the same emission factors, and results were compared with the baseline case by calculating the relative variation in total carbon footprint.

Table 2 summarizes the parameter variations considered in the sensitivity analyses; the results of the sensitivity analysis were used to identify the parameters with the greatest impact on the system’s carbon footprint and to define the improvement scenarios analysed in the subsequent scenario analysis.

### 2.6. Scenario Analysis

The scenario analysis was designed to evaluate realistic industrial improvement strategies rather than theoretical extreme conditions. The selected scenarios combine the parameters identified as the most influential in the sensitivity analysis and represent technically feasible interventions, including increased recycled polypropylene content, renewable electricity integration and process optimisation. Consequently, the scenarios provide a practical assessment of the achievable carbon footprint reduction under realistic industrial conditions. Accordingly, the scenarios do not represent extreme configurations but rather plausible combinations of measures that can be implemented in the short to medium term [23]. For each scenario, mass and energy balances were updated; consequently, the life cycle inventory was modified, and total CO_2_-equivalent emissions were recalculated using the same emission factors and methodological assumptions adopted for both the baseline case and the sensitivity analysis.

## 3. Results

### 3.1. Life Cycle Inventory

Table 3 reports the life cycle inventory (LCI) of the representative model, normalized to the functional unit of 1 ton of finished product. The inventory includes all material, energy, water, and waste flows within the cradle-to-gate system boundaries and provides the basis for greenhouse gas emission calculations and subsequent analyses.

Table 4 presents the baseline carbon footprint results, expressed as total CO_2_-equivalent emissions and disaggregated by process stage and emission category according to ISO 14064-1. The total carbon footprint of the baseline configuration amounts to 1.44 tCO_2_e per ton of finished product. The largest contributions are associated with energy-intensive processing stages, particularly the material purification phase, followed by shaping extrusion and injection moulding.

From an emission category perspective, indirect emissions from purchased electricity (Category 2) and waste treatment (Category 4.3) account for most of the total impacts. In contrast, emissions related to purchased goods and auxiliary materials (Category 4.1) contribute to a lesser extent.

The baseline carbon footprint of 1.4365 tCO_2_e/t obtained in this study reflects a specific industrial configuration based on recycled polypropylene. The baseline aligns with the broader scientific consensus that replacing virgin inputs with secondary post-consumer polyolefins lowers upstream manufacturing impacts [4,7,24].

However, a detailed breakdown by emission categories reveals significant trade-offs:

- Indirect emissions from purchased electricity (Category 2) constitute the second largest contribution (44.4% of the total). Within the boundaries of the facility, the combined electricity consumption of shaping extrusion and injection moulding represents the primary operational hotspot: high reliance on the electric grid mix for thermoplastic transformation phases is coherent with other scientific results, in which core manufacturing hotspots are due to the high energy demands of heating and shaping units [25,26].
- Indirect emissions from purchased goods and services (Category 4.1) contribute to a lesser extent. This minimal contribution represents a major point of divergence from conventional studies: in virgin PP manufacturing chains, the extraction of fossil feeds and monomer production constitute the overwhelming environmental hotspot, often exceeding 90% of the cradle-to-gate impact [25]. Conversely, our study utilizes sorted waste as the core input, which carries a significantly lower carbon background compared to the substantially higher upstream emissions associated with virgin fossil feedstocks [7].
- Indirect emissions from waste treatment (Category 4.3) represent the largest overall contribution to the baseline case (51.1% of the total), predominantly driven by the material purification stage. This finding highlights a critical trade-off: while mechanical recycling avoids high-impact fossil resource extraction, it shifts a non-negligible portion of the environmental burden onto early-stage conditioning and purification processes at the plant level. This aspect is often underrepresented in scientific literature and in generic datasets of plastic processing [27,28].

### 3.2. Sensitivity Analysis Results

**Figure 2:**
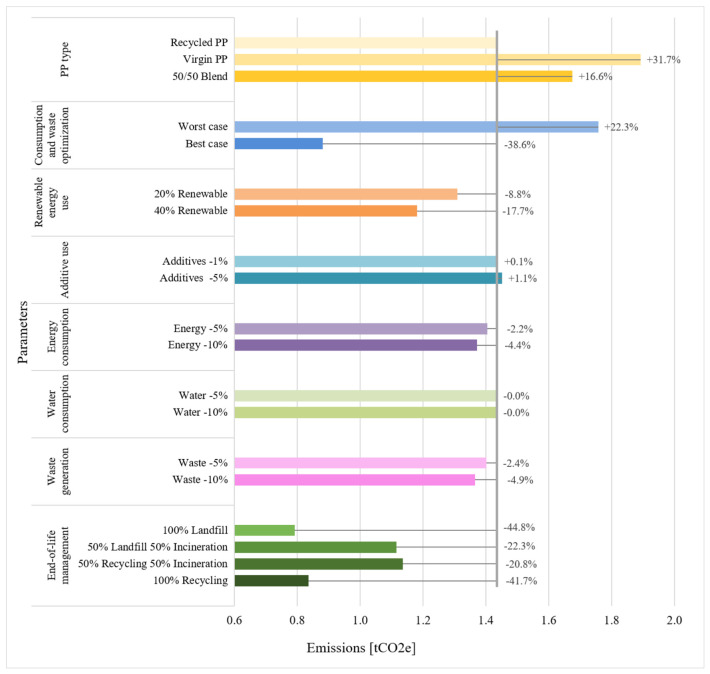
Sensitivity of the carbon footprint to key parameters, expressed in tCO_2_e.

The sensitivity analysis evaluates the influence of key parameters on the carbon footprint of PP processing by quantifying the relative variation in total tCO_2_e emissions compared to the baseline case. Results are expressed in tCO_2_e to allow a consistent comparison across parameters. Figure 2 presents the variation in total carbon footprint associated with each analysed parameter. The sensitivity cases are grouped by parameter category, and the vertical grey line represents the carbon footprint of the baseline configuration (1.4365 tCO_2_e per tonne of finished product; Table 4). The percentage variation relative to the baseline is reported for each case to facilitate comparison.

As shown in Figure 2, the type and origin of polypropylene exert the strongest influence on the system. The use of 100% virgin polypropylene leads to a significant increase in emissions (+31.8%), while a 50/50 blend of virgin and recycled material results in a moderate increase (+16.7%) compared to the baseline. These results confirm the dominant role of upstream material production in determining the overall carbon footprint.

Operational performance also has a substantial impact on emissions. The adoption of best-case conditions leads to a reduction of −38.7%, whereas worst-case conditions result in an increase of +22.4%. This wide variation reflects the influence of process performance and operational variability across different industrial configurations.

Changes in the electricity supply mix produce consistent effects. Increasing the share of renewable electricity to 20% and 40% leads to emission reductions of −8.9% and −17.7%, respectively, confirming the relevance of energy sourcing in energy-intensive processes.

Improvements in process efficiency parameters lead to more moderate emission reductions. A decrease in energy consumption of 5% and 10% results in reductions of −2.2% and −4.4%, respectively, while reducing waste generation by 5% and 10% leads to reductions of −2.4% and −4.9%. In contrast, variations in water consumption and additives use have a negligible influence on the overall carbon footprint, with changes below 1% across all analysed conditions. This indicates that these parameters play a limited role compared to other factors influencing climate change impacts within the defined system boundaries.

Finally, waste management options exhibit a wide range of outcomes. Incineration is associated with the highest emissions, whereas landfill and recycling scenarios result in lower impacts within the cradle-to-gate system boundaries. The slightly higher emissions associated with recycling compared to landfill are due to the exclusion of avoided burden credits for secondary material production within the defined system boundaries.

### 3.3. Scenario Analysis Results

A scenario analysis was performed to evaluate the combined effect of multiple parameters under realistic operating conditions. While sensitivity analysis isolates the influence of individual variables, the scenario analysis reflects plausible industrial configurations by simultaneously modifying key parameters. The analysed scenarios are derived from combinations of the most relevant parameter variations identified in the sensitivity analysis and aim to assess potential strategies for reducing the carbon footprint of polypropylene processing, including changes in material composition, energy sourcing and process performance.

The following scenarios were evaluated and compared:

- Baseline scenario: average operating conditions derived from industrial datasets, national electricity mix and incineration of process waste.
- Scenario 1—Material and energy supply improvement: combination of favourable operational conditions and electricity supply identified in the sensitivity analysis (A1.2 and A2.2), representing best-case conditions and a partially decarbonized electricity mix (60% national grid and 40% renewable). This scenario represents a strategy primarily focused on process efficiency and energy sourcing.
- Scenario 2—Process optimisation: combination of improvements in key process efficiency parameters (B1.2, B2.2 and B3.2), including a 10% reduction in energy consumption, water use and process waste generation. This scenario represents a strategy centered on operational efficiency and process control.
- Scenario 3—Waste management improvement: combination of reduced process waste generation and improved end-of-life management (B3.2 and C3.4), including a 10% reduction in waste generation and 100% recycling of plastic process residues. This scenario represents the most favourable configuration from a circular economy perspective.

**Figure 3:**
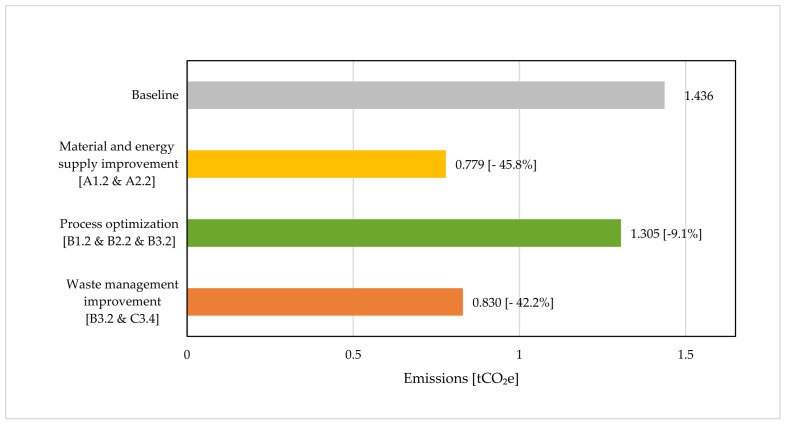
Carbon footprint comparison of baseline and improvement scenarios.

Figure 3 compares the total carbon footprint of the three improvement scenarios with the baseline configuration, which is consistently represented in grey.

All scenarios result in a reduction of emissions compared to the baseline. Scenario 1 shows the most significant decrease (−45.8%), highlighting the dominant role of process performance and energy supply. Scenario 2 leads to more moderate reductions (−9.1%), as it is based on improvements in process efficiency, including reductions in energy consumption, water use and waste generation. Scenario 3, combining waste reduction and full recycling of process residues, also achieves a substantial decrease (−42.2%) relative to the baseline, confirming the relevance of circular strategies for managing process waste. Taken together, the results show that combining improvements across multiple parameters leads to significantly greater emission reductions than isolated interventions.

## 4. Discussion

The results of this study provide relevant insights into the environmental performance of PP processing based on primary industrial data. The analysis shows that upstream material production is the dominant contributor to the overall carbon footprint, with the choice between recycled and virgin polypropylene representing the most influential parameter. This trend is consistent with previous LCA studies on plastic systems, where feedstock and monomer production are typically identified as the main environmental hotspots [1,3]. A second key factor is operational variability, as evidenced by the wide differences observed between best-case and worst-case industrial configurations. The magnitude of these variations suggests that differences in equipment efficiency, process control, material utilisation and process scrap generation can significantly affect the carbon footprint of plastic processing. This finding is particularly relevant from an industrial perspective, as it indicates that substantial emission reductions can be achieved without structural process changes, but rather through improved operational practices and equipment optimisation. This pattern aligns with the broader literature on plastic waste management and circular economy strategies [25].

Electricity supply also plays a key role, particularly in energy-intensive processes such as extrusion and injection moulding [3]. Partial decarbonisation through the integration of renewable energy leads to consistent and measurable emission reductions in both sensitivity and scenario analyses. Although the renewable shares considered in this study are relatively conservative, the results indicate that energy sourcing represents a robust and scalable lever for emission reduction in plastic processing systems. Increasing the share of renewable electricity consistently reduces emissions, underscoring the importance of energy decarbonisation in industrial contexts.

In contrast, other parameters often considered in environmental improvement strategies show a limited influence on the overall carbon footprint. Variations in water consumption and additive use result in negligible emission changes within the system boundaries considered. This suggests that, from a climate change perspective, interventions focusing exclusively on these aspects are unlikely to deliver significant benefits, although they may remain relevant for other environmental impact categories.

The analysis of waste management options provides additional insights, although results must be interpreted carefully due to the adopted system boundaries. The slightly higher emissions associated with recycling compared to landfill are a consequence of excluding avoided burden credits for secondary material substitution. In this study, recycling processes are accounted for based on their direct emissions only, while the potential environmental benefits associated with substituting virgin polypropylene with recycled material are not credited within the system boundaries. This modelling choice reflects a cradle-to-gate perspective focused on the processing stage. As a result, the benefits of recycling are not fully captured in the reported results, leading to a conservative estimation of its environmental performance. This highlights the importance of methodological choices in LCA studies and underlines the need for transparent reporting of assumptions. From a circular economy perspective, the results emphasize the importance of integrating recycled materials into production systems while simultaneously improving process efficiency and energy sourcing.

Although the use of primary industrial data substantially improves the reliability of the inventory compared with generic LCA databases, several limitations should be acknowledged. First, the dataset includes only four industrial facilities located in Italy and Austria, which limits the geographical representativeness of the study. Electricity generation, industrial operating conditions, and, to a lesser extent, waste management modelling may vary considerably across Europe and worldwide. Consequently, the absolute carbon footprint values reported here should not be interpreted as universally representative of polypropylene processing. Furthermore, the analysed production lines mainly represent extrusion-based processing followed by injection moulding. Other processing technologies, including blow moulding, thermoforming, fibre production and rotational moulding, were outside the scope of this work and may present different environmental profiles. Another source of uncertainty arises from the use of secondary life cycle inventory data for virgin polypropylene production. Although these datasets are widely adopted in LCA practice, differences between database inventories and specific industrial production routes may influence the comparison between virgin and recycled material options. Finally, while primary data were available for additive quantities, detailed compositional information for the additives was not available, as the participating companies did not disclose this proprietary information. Consequently, additive-related emissions were modelled using representative literature-based emission factors. Although this approach is consistent with current practice and known inventory limitations for plastic additives [10], it introduces an additional source of uncertainty that should be considered when interpreting the absolute carbon footprint values.

Despite these limitations, the results provide a robust basis for identifying the relative contribution of the main emission sources and for supporting industrial decision-making aimed at reducing greenhouse gas emissions.

## 5. Conclusions

This study presents a cradle-to-gate life cycle assessment of polypropylene processing based on primary industrial data collected from operating manufacturing facilities. By combining primary inventory data with sensitivity and scenario analyses, the study addresses a limitation of many previous LCAs of polypropylene processing, which are predominantly based on generic secondary datasets. The proposed approach provides a more industry-relevant assessment of industrial processing conditions while identifying the operational parameters that most strongly influence greenhouse gas emissions.

The results demonstrate that raw material selection is the dominant contributor to the overall carbon footprint, with the use of recycled polypropylene representing the most effective mitigation strategy. Process optimisation and the progressive decarbonisation of electricity supply provide additional opportunities for emission reduction, whereas other operational parameters have a comparatively smaller influence within the analysed system boundaries.

Beyond the specific industrial case studies analysed, the findings demonstrate the importance of integrating primary industrial data into life cycle assessments of plastic processing. Rather than providing universally applicable carbon footprint values, the study identifies robust emission hotspots and operational priorities that can support industrial decision-making and the development of more representative environmental inventories for polypropylene conversion processes.

Future research should expand the industrial dataset to include a broader range of production technologies (e.g., blow moulding, thermoforming and fibre production) and facilities operating under different geographical and electricity supply conditions in order to evaluate the transferability of the identified emission drivers. Improved availability of formulation-specific inventory data for additives would further reduce modelling uncertainty, while extending the assessment beyond the cradle-to-gate boundary would enable the quantification of the environmental benefits associated with circular material flows and end-of-life management.

Overall, this study demonstrates that the combination of primary industrial data and transparent life cycle modelling provides a robust framework for identifying practical decarbonisation opportunities in polypropylene processing and may support both industrial decision-making and the development of future sector-specific LCA inventories.

## Figures and Tables

**Table 2:** Definition of sensitivity analysis cases and scenario parameters.

Objective	Parameters
PP type	100% recycled PP pellets
	100% virgin PP pellets
	50% virgin PP and 50% recycled PP
Optimisation of consumption and waste reduction	Average case
	Worst case
	Best case
Use of renewable energy sources	100% grid mix
	80% grid mix and 20% renewable
	60% grid mix and 40% renewable
Reduction in additive use	Additives unchanged
	Additives reduced by 1%
	Additives reduced by 5%
Reduction in energy consumption	Energy use unchanged
	Reduce energy use by 5%
	Reduce energy use by 10%
Reduction in water consumption	Water use unchanged
	Water use reduced by 5%
	Water use reduced by 10%
Reduction in waste generation	Waste generation unchanged
	Waste reduced by 5%
	Waste reduced by 10%
Changes in process waste end-of-life	100% incineration
	100% landfill
	50% landfill and 50% incineration
	50% recycling and 50% incineration
	100% recycling

**Table 3:** Life cycle inventory of the representative system.

		INPUT						OUTPUT						
	Sorted Waste [kg]	Plastic Product [kg]	CaCO_3_ [kg]	Additive [kg]	Mineral Oil [kg]	Water [L]	Energy [kWh]	Wastewater [L]	Water [L]	Plastic Product [kg]	Sludge [kg]	Plastic Losses [kg]	Metals [kg]	Exhausted Oil [kg]
Purification	1015.96					1920.17	121.92	1937.18		752.57	115.82	120.41	10.16	
Compounding extrusion		752.57				22.58	245.86		22.58	714.94		37.63		
Mixing		714.94					2.8 × 10^−6^			707.41		7.53		
Shaping extrusion		707.41	326.12	10.98	0.02	28.61	1082.59	0.00	28.61	1025.97		18.55		0.02
Injection moulding		1025.97			2.99	28.00	1245.40	0.18	27.82	1000		25.97		2.99

**Table 4:** CO_2_-equivalent emissions of the baseline case.

Process Step	Total Emissions [tCO_2_e]	Category 2.1 Emissions [tCO_2_e]	Category 4.1 Emissions [tCO_2_e]	Category 4.3 Emissions [tCO_2_e]
Purification	0.5009	0.0288	0.0156	0.4565
Compounding extrusion	0.1742	0.0581	0.0000	0.1161
Mixing	0.0232	0.0000	0.0000	0.0232
Shaping extrusion	0.3595	0.2558	0.0464	0.0572
Injection moulding	0.3787	0.2943	0.0042	0.0802
Total	1.4365	0.6371	0.0662	0.7333

## Data Availability

The datasets presented in this article are not readily available due to non-disclosure agreements (NDAs) with partner companies. Requests to access the datasets should be directed to the corresponding author and are subject to third-party authorization. The raw data supporting the conclusions of this article are available from the authors upon reasonable request, subject to NDA restrictions.

## Abbreviations

The following abbreviations are used in this manuscript:

LCA	Life Cycle Assessment
LCI	Life Cycle Inventory
PP	Polypropylene
